# Interest of retro-anal levator plate myorrhaphy in selected cases of descending perineum syndrome with positive anti-sagging test

**DOI:** 10.1186/1471-2482-8-13

**Published:** 2008-07-30

**Authors:** Jacques Beco

**Affiliations:** 1 University of Liège, Department of Gynaecology, CHU Notre Dame des Bruyères, Rue de Gaillarmont 600, B-4032 Chênée, Belgium; 2 CHC Clinique Sainte Elisabeth, Rue du Naimeux, B-4802 Heusy, Belgium

## Abstract

**Background:**

Levator plate sagging (LPS), usually called descending perineum syndrome, is one of the main defects encountered in perineology. This defect is classically associated with colo-proctologic functional troubles (dyschesia and anal incontinence) but can also induce perineodynia, gynaecological and lower urinary tract symptoms.

**Methods:**

A retrospective case series of nine female patients (mean age: 44.3) underwent an isolated retro-anal levator plate myorrhaphy (RLPM) to treat symptomatic LPS confirmed by rectal examination and/or Perineocaliper^®^. An anti-sagging test (support of the posterior perineum) must significantly improve the symptoms that were resistant to conservative treatment. The effect of the procedure on the symptoms of the 3 axes of the perineum (urological, colo-proctologic and gynecological) and on perineodynia was evaluated during a follow up consultation more than 9 months after surgery. The effect of RLPM on the position of the anal margin and on the levator plate angle was studied using rectal examination, Perineocaliper^® ^and retro-anal ultrasound.

**Results:**

Before surgery, anti-sagging tests were positive for dyschesia, urinary urgency and pain. After a mean follow-up of 16.1 months, RLPM resolved or improved 2/2 cases of stress urinary incontinence, 3/5 of urinary urgency, 3/4 of dysuria, 3/3 of anal incontinence, 7/8 of dyschesia, 3/4 of cystocele, 4/5 of rectocele, 5/8 of dyspareunia and 6/7 of perineodynia. Rectal examination showed a complete suppression of sagging in 4 patients and an improvement in the 5 others. The mean reduction of perineal descent was 1.08 cm (extremes: 0–1.5). Using retro-anal ultrasound of the levator plate, the mean reduction of sagging was 12.67 degrees (extremes: 1 – 21).

**Conclusion:**

Anti-sagging tests can be used before surgery to simulate the effect of RLPM. This surgical procedure seems to improve stress urinary incontinence, frequency, nocturia, urgency, dysuria, anal incontinence, dyschesia, dyspareunia, perineodynia, cystocele and rectocele. These results must be confirmed by a larger case series.

## Background

Perineology is a new speciality that deals with the functional troubles of the three axes of the perineum [1-4]. This interdisciplinary and holistic field is approached from the angles of anatomy, biomechanics and physiology, avoiding at all costs any side effects (*primum non nocere*). There are seven basic, defect specific, useful surgical procedures that apply in perineology [2]. Retro-anal levator plate myorrhaphy (RLPM) is dedicated to treating the "levator plate sagging" defect.

The usual name for this levator plate sagging is descending perineum syndrome (DPS). This syndrome is well described by Parks in 1966 [5]. For this author this title is mainly descriptive, as perineal descent on straining is both the cause of symptoms and the most obvious physical sign.

For Parks, the main symptoms of DPS are dyschesia (partial and intermittent obstruction by the anterior rectal wall), pain (dull aching pain in the perineum or sacrum after defecation), bleeding or passage of mucus (prolapse of the anterior rectal wall) and anal leakage. The physical signs of this syndrome on external examination are a low position of the anus at rest or a perineal descent on straining (more than 3 cm). During this straining, the anal mucosa may pout. On rectal examination, during straining, the pubo-rectalis descends sharply and the anterior rectal wall pushes down on the examining finger. Muscle tone is easily overcome by posterior traction.

For Parks, the first step in the treatment of this syndrome consists of preventing further damages by avoiding straining during defecation and emphasizing pelvic floor reeducation.

For the most significant cases with rectal prolapse, Parks has developed a new surgical procedure called "post-anal perineorrhaphy" [6]. This procedure, also known as "post-anal repair", has been used by many other authors to treat faecal incontinence [7-13].

In 1982, Nichols used a "retro-rectal levatorplasty" to treat an uncommon type of genital prolapse characterized by descent of the anus and sagging of the levator plate associated with severe constipation [14].

In 1987, Shafik presented his experience with "levatorplasty" in the treatment of complete rectal prolapse [15].

To improve the process of defecation by reducing levator plate sagging, Nichols [14,16] proposed using a special toilet seat with a small opening and Lesaffer [17,18] created a "perineum device" to support the perineum. These proposals were the first "anti-sagging tests".

More recently, Beco [19] demonstrated that besides dyschesia and anal incontinence, a perineal descent of more than 1.5 cm, measured with a Perineocaliper^® ^during straining in the gynaecological position (with thighs flexed to 90 degrees), significantly increased the frequency of urinary incontinence, dyspareunia, dysuria, cystocele and rectocele. The frequency of the 3 clinical signs of pudendal neuropathy [20] was also significantly increased.

**The first aim **of this study is to show the diagnostic importance of "anti-sagging tests" on the symptoms of dyschesia, dysuria, dyspareunia, urinary incontinence, urgency and perineodynia experienced in levator plate sagging while standing. These tests can be performed by the patient herself or by the examining practitioner.

**The second aim **is to evaluate the effect of a simplified retro-anal levator plate myorrhaphy (RLPM) on different symptoms and on perineal position and descent during straining.

## Methods

### 1. Studied population

Between March 2000 and January 2007, 104 retro-anal levator plate myorrhaphies (RLPM) were performed to treat levator plate sagging. To study the effect of RLPM, only 9 cases of isolated procedures were taken into account. The mean follow up was 16.1 months (extremes: 10–39 months). The mean age of patients was 44.3 years (extremes: 29–63) and mean parity 2.11 (extremes: 1–3). Five patients had a history of abdominal hysterectomy and 2 of surgery for prolapse. Five patients had difficult deliveries (2 vacuum extractors and 3 forceps). The approval for this study was granted by the CHC – Clinique Sainte Elisabeth Ethic Committee and each patient signed a written informed consent before surgery.

### 2. Pre-operative evaluation

In addition to the classical history and clinical examination of the three axes of the perineum (urological, gynecological and colo-proctologic), special attention was given to diagnose pudendal neuropathy and levator plate sagging.

#### History

##### -Urinary symptoms

Stress and urge urinary incontinences are evaluated according to a 4 level ordinal scale depending on the amount of the leakage: 0 = no incontinence, 1 = mild incontinence (few drops of urine), 2 = moderate incontinence (moderate amount) and 3 = severe incontinence (large amount). The number of pads used per day is included.

Daily frequency is based on the mean time (in minutes) between 2 micturitions. It is considered abnormal if this time is less than 90 minutes.

Nocturnal frequency is evaluated by the number of micturitions during the night. The patient suffers from nocturia if there is more than 1 micturition per night.

Urgency was evaluated according to a 3 level ordinal scale: 0 = no urgency, 1 = occasional urgency, 2 = constant urgency.

Dysuria is evaluated with the same 3 level ordinal scale.

##### - Colo-proctologic symptoms

For anal incontinence a four level ordinal scale was used: 0 = no incontinence, 1 = gas incontinence, 2 = liquid incontinence, 3 = solid incontinence.

The patient presents dyschesia if the defecation process is abnormal, including formation of plugs, the need for enemas, glycerin suppositories or digital manipulation to evacuate stool. The importance of dyschesia has been evaluated according to a three level scale: 0 = no dyschesia, 1 = occasional dyschesia and/or mild difficulties to defecate, 2 = continuous or severe.

##### - Dyspareunia and other perineodynia

The importance of dyspareunia has been evaluated according to a 3 level scale: 0 = no dyspareunia, 1 = mild dyspareunia, 2 = severe dyspareunia.

For perineodynia (perineal pain), the intensity of pain is evaluated using a classical visual analog scale going from 0 to 10. The different characteristics of pain are also studied.

#### Clinical examination

The entire examination is done in gynecological position (with the thighs flexed to 90 degrees).

##### - Basic examination

Cystocele, rectocele, enterocele and uterus descent have been graded from 0 to 3 according to the French classification [21,22] during Valsalva's maneuver and with a speculum moving away the vaginal wall in front of the prolapse (0 = no descent, 1 = in the vagina, 2 = at the level of vulvae skin, 3 = outside the vagina).

In case of dyspareunia or perineodynia, the most prominent painful areas are explored by vaginal and rectal examination.

##### - Pudendal neuropathy

The three clinical signs of pudendal neuropathy (abnormal pinprick sensibility, pain over the pudendal nerve during rectal examination and positive skin rolling test) were searched in the 9 patients [20,23].

##### - Levator plate sagging

To evaluate levator plate sagging, we have used 3 methods: rectal examination, the Perineocaliper^® ^and retro-anal ultrasound.

During **rectal examination**, the position of the levator plate is evaluated with the index finger at rest and during Valsalva's maneuver. The two first phalanges of this finger are in the rectum in close contact with the levator plate. During straining, a small amount of traction on the finger is used. A three levels ordinal scale is used: 0 = no sagging of the levator plate (90° angle between anal canal and levator plate plane), 1 = moderate sagging of the levator plate (between 0 and 2) and 2 = complete levator plate sagging (180° angle between anal canal and levator plate plane).

**Figure 1:**
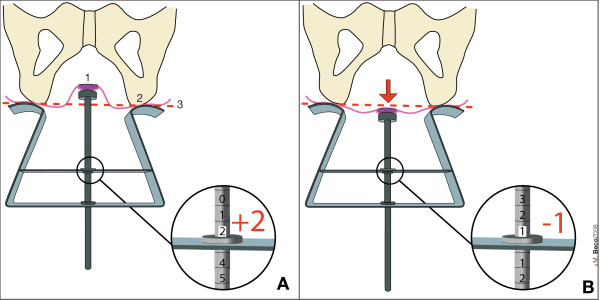
**Use of Perineocaliper^® ^(Duchateau SA, Liège, Belgium)**. A: position of anal margin 2 cm higher than the ischial tuberosities (value = + 2 cm) at rest and in gynecological position (with thighs flexed to 90 degrees). B: during Valsalva's maneuver (red arrow) the anal margin is 1 cm below the ischial tuberosities (value = - 1 cm). View from the top as during clinical measurement. In this case, perineal descent = 3 cm (difference between A and B). 1 = anal margin. 2 = ischial tuberosity. 3 = level of the ischial tuberosities = reference or zero level.

The **Perineocaliper**^® ^(Duchateau SA, Liège, Belgium) has been developed to evaluate the position of anal margin with respect to the ischial tuberosities at rest and during a Valsalva's maneuver in the gynecological position (with thighs flexed to 90 degrees) (Figure 1).

If the anal margin is located higher than the ischial tuberosities the value is positive. If it is located below, the value is negative. Perineal descent corresponds to the difference between the position at rest and during straining.

**Figure 2:**
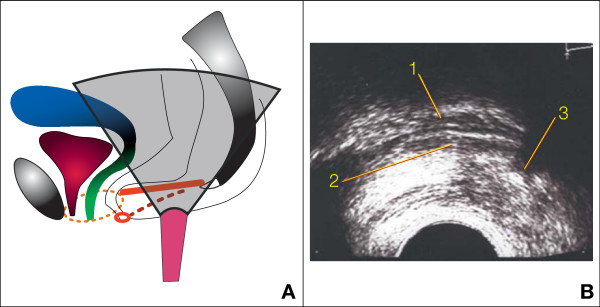
**Retro-anal ultrasound of the levator plate**. A: position of the probe between anus and coccyx (levator plate or ano-coccygeal raphe in red). B: ultrasonographic image: 1 = posterior rectal wall, 2 = levator plate, 3 = coccyx. Dotted lines represent structures which are not in the section plane: thin lines = limits of the levator hiatus, thick line = right pudendal nerve. Small red ring = anal sphincter.

**Retro-anal ultrasound **has been done with an end-fire transvaginal probe (Hitachi^®^) emitting at 6.5 MHz [24] The patient is lying in gynecological position (with the thighs flexed to 90 degrees). The probe must be perfectly horizontal with its tip located in the midline 1 or 2 cm in front of the coccyx to obtain a sagittal section (Figure 2).

The practitioner has to be very cautious to avoid any lifting or supportive effect of the levator plate with the probe at rest and during straining. The angle between the levator plate ("ano-coccygeal raphe" in the midline) and the vertical plane has been measured at rest and during Valsalva's maneuver. The angle of sagging corresponds to the difference between these 2 values.

##### - Anti-sagging tests

**Figure 3:**
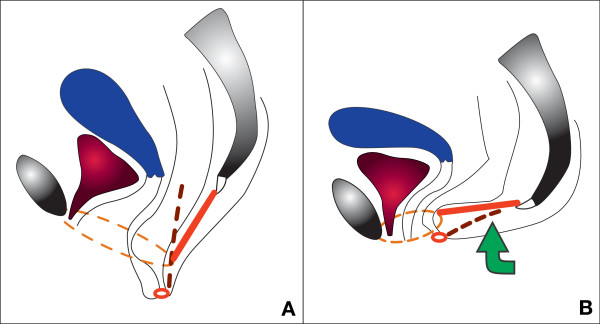
**Anti-sagging test on a sagittal section**. A: sagging of the levator plate – descending perineum syndrome (levator plate or ano-coccygeal raphe in red) during Valsalva's maneuver. B: anti-sagging test: support of the posterior perineum restores normal position. Dotted lines represent structures which are not in the section plane: thin lines = limits of the levator hiatus (increase of its size if perineal descent during Valsalva's maneuver), thick lines = right pudendal nerve (stretching induced by perineal descent). Small red ring = anal sphincter.

The aim of the "anti-sagging tests" is to reduce the sagging of the levator plate, which simulates the effect of retro-anal levator plate myorrhaphy (Figure 3).

For dyschesia and dysuria, the patient has to move back as much as possible on the toilet seat during defecation and micturition to support the levator plate (between coccyx and anus).

For dyspareunia, if vaginal examination reproduces the classical pain induced by intercourse (usually at the level of the pubo-rectalis muscle, utero-sacral ligaments, transverse muscle or vaginal scar), the test consists of lifting the posterior perineum (between coccyx and anus) with two or three fingers and to evaluate the effect on pain. The patient can also try this test during intercourse (or use sexual positions where the buttocks are higher than the head).

The anti-sagging test can be tried by the practitioner and taught to the patient while standing for urinary urgency or perineodynia. During urodynamic exploration, it is possible to try the effect of this test on the urgent need to urinate, on bladder capacity or even on urinary stress incontinence.

The "anti-sagging test" is positive if there is a very clear improvement of the symptom studied during this maneuver.

### 3. Indication for surgery

When diet, drugs and physiotherapy fail, surgery is indicated when the anti-sagging test dramatically improves the resistant symptoms associated with complete levator plate sagging on rectal examination (180° angle between anal canal and levator plate plane) and/or a perineal descent of more than 1 cm (measured with the Perineocaliper^®^).

### 4. Surgical procedure

During the 48 hours before surgery, the patient has to eat a residue free diet and must take paromomycine 2 g per day. To complete the intestinal preparation, an enema is done the evening before surgery.

**Figure 4:**
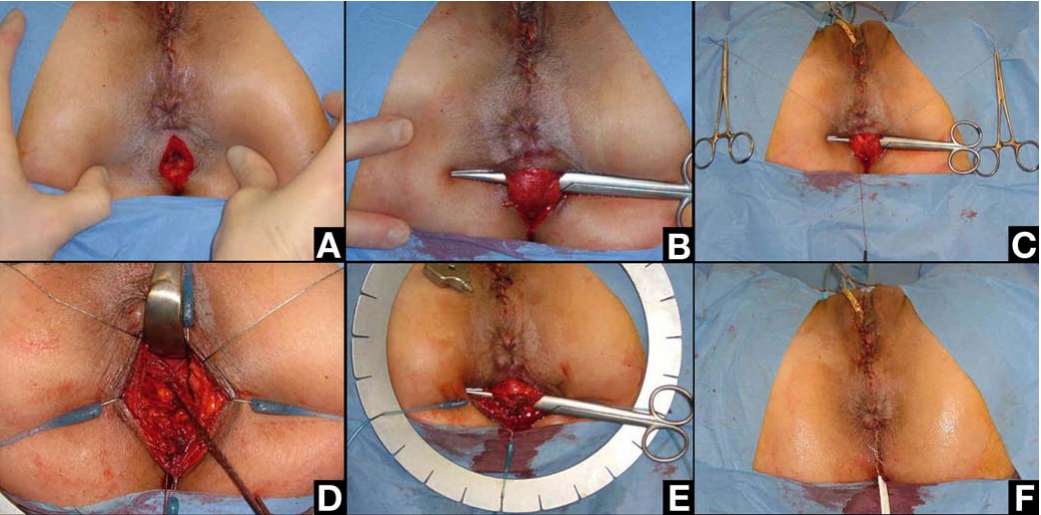
**Retro-anal levator plate myorrhaphy: surgical steps**. A: Skin incision. B: Scissors introduced between levator plate and ano-coccygeal ligament (also called intermediate loop of the external anal sphincter). C: The ano-coccygeal ligament ready to be cut (two extremities marked with a thread). D: Levator plate myorrhaphy between coccyx and ano-rectal junction until suppression of the sagging (checked by rectal examination). E: Ano-coccygeal ligament repaired. F: Skin closure with a Y-shaped multi-tubular drain at the posterior edge of the incision.

The patient is installed in a gynecological position with hyperflexion of the thighs. The surgical procedure begins with a sagittal incision 4 cm long between anus and coccyx (Figure 4a). The two ischio-rectal fossae are opened with the tip of the scissors laterally to the ano-coccygeal ligament (also called "intermediate loop of the external anal sphincter" [25]). The space between the ano-coccygeal ligament and the levator plate is opened with the finger. The scissors are passed from left to right over the ano-coccygeal ligament to isolate this ligament (Figure 4b). It is cut after having marked its two extremities with a suture (Figure 4c).

The levator plate myorrhaphy began in front of the coccyx. Before putting stitches, it is necessary to "clean" the muscle with the finger by removing the adherent grease as much as possible. This step is necessary to be sure to put the stitches into the muscle. The first stitch is very important. It is located just in front of the coccyx. It must take a good bit (1 cm; with a dexon^® ^2, needle GS-21) of the levator plate on each side to tighten the muscle but without excessive tension not to tear it. Traction on this stitch to the rear checks the solidity of this first point of myorrhaphy and presents the two edges of the levator plate to be sutured. Usually two to four stitches of dexon^® ^2 (with some "figure of eight" if possible) are necessary to suppress completely the sagging (Figure 4d). To avoid rectal injury, the stitches can be put with a finger in the rectum to check the position of the needle. The last stitches must remain behind the level of the anal canal to avoid damage to the rectal branches of the pudendal nerves.

At the end of the procedure, rectal examination confirms the absence of sagging of the levator plate (90° angle between anal canal and levator plate plane). The ano-coccygeal ligament is repaired with two to three stitches of dexon^® ^2 (Figure 4e). A Y-shaped multi-tubular drain is put into the two ischio-rectal fossae and exits through the posterior edge of the initial incision. The subcutaneous tissue is then closed followed by closure of the skin with simple stitches of vicryl rapide^® ^2.0 (Figure 4f). The drains are usually removed the 5^th ^day after surgery. Antibiotic coverage is continued for 5 days.

After surgery, the patient is told to avoid heavy lifting, constipation (use of soft laxatives), and chronic cough and to move back on the toilet seat to support her posterior perineum during defecation and voiding, for a minimum of two months and ideally for the rest of her life.

### 5. Evaluation of the procedure

The effects of RLPM have been evaluated more than 9 months after surgery by using the same history, clinical examination and retro-anal ultrasound as before the operation.

## Results

### 1. Anti-sagging tests before surgery

Besides a clear levator plate sagging, a positive anti-sagging test on a severe perineal symptom is crucial in the indication of a RLPM. The results of the pre-operative anti-sagging tests of this case series are presented in Table 1.

**Table 1:** Results of the anti-sagging tests before surgery

		During clinical examination		Subjective
			
Patients	Follow-up (months)	Pain	Urgency	Dyschesia
Ck	12	Improved	-	-
Dz	18	- (**)	-	Improved
Rx	12	Improved	-	-
Re	19	Improved	Improved(*)	-
Hy	39	Improved	-	-
Ro	11	Improved	-	-
Ns	12	Improved	-	Improved
Sn	12	Improved	-	Improved
Hs	10	Improved	-	Improved

The test is positive if the symptoms are significantly improved.

(*) during bladder filling.

(**) – = not done.

No test for dysuria and stress urinary incontinence done in this population.

### 2. Effect on symptoms of the three axes of the perineum and on perineodynia

#### Urological axis (Table 2)

**Table 2:** Effect of RLPM on the urological axis

Patients	Follow-up (months)	SUI (0–3)		Frequency (min)		Nocturia (nr/night)		Urgency (0–2)		Dysuria (0–2)	
						
		Before	After	Before	After	Before	After	Before	After	Before	After
Ck	12	0	0	60	180	2	0	0	0	2	0
Dz	18	1	0	120	120	0	0	0	0	0	0
Rx	12	0	0	180	180	0	0	0	0	0	0
Re	19	0	0	60	180	0	0	2	0	0	0
Hy	39	0	0	120	120	1	1	1	1	0	0
Ro	11	2	0	240	240	0	0	0	0	0	0
Ns	12	0	0	120	120	1	0	2	0	2	1
Sn	12	0	0	120	180	1	1	1	0	1	0
Hs	10	0	0	120	180	0	1	1	1	1	1

SUI = stress urinary incontinence

min = minutes

In this case series no patient had urge incontinence

In this case series, no patients had urge incontinence. The 2 cases of stress urinary incontinence were cured (one used 1 pad per day and the other 3 pads per day). The 2 cases with frequency and the one with nocturia were cured. Dysuria was cured in 2 patients, improved in one and was unchanged in the other one. Urgency was cured in 3 patients and unchanged in two.

#### Colo-proctologic axis (Table 3)

**Table 3:** Effect of RLPM on the colo-proctologic axis

		Anal incontinence (0–3)		Dyschesia (0–2)	
			
Patients	Follow-up (months)	Before	After	Before	After
Ck	12	0	0	2	0
Dz	18	2	1	2	0
Rx	12	0	0	2	0
Re	19	1	0	2	0
Hy	39	0	0	2	2
Ro	11	0	0	0	0
Ns	12	0	0	2	1
Sn	12	1	0	2	0
Hs	10	0	0	2	0

Two cases of gas incontinence were cured and one of liquid incontinence improved. Dyschesia was completely cured in 6 patients, improved in one and was unchanged in one case.

#### Gynecological axis (Table 4)

**Table 4:** Effect of RLPM on the gynecological axis

		Dyspareunia (0–2)		Vaginal examination (location of pain)		Cystocele (0–3)		Rectocele (0–3)	
					
Patients	Follow-up (months)	Before	After	Before	After	Before	After	Before	After
Ck	12	2	0	Left PR (right absent)	Reduced	0	0	0	0
Dz	18	0	0	-	-	0	0	1	0
Rx	12	2	0	Right PR Left scar	Reduced	0	0	0	0
Re	19	2	N.I.	PR bilateral	0	1	1	1	0
Hy	39	2	2	Right PR	0	0	0	0	0
Ro	11	2	0	Vaginal cuff	0	1	0	1	0
Ns	12	2	N.I.	PR bilateral	Vaginal cuff	1	0	1	1
Sn	12	2	1	Left PR	Reduced	0	0	0	0
Hs	10	2	0	US ligaments	0	2	0	1	0

PR = pubo-rectalis

US = utero-sacral

N.I. = no intercourse (other reason than pain)

Dyspareunia was cured in 4 patients, improved in one and was unchanged in one. Two patients did not have intercourse after surgery for other reasons than pain. Three cystoceles were cured (two grade 1 and one grade 2). One was unchanged. Four grade 1 rectocele disappeared and one was unchanged. Pain during vaginal examination disappeared in 4 patients and was reduced in another four.

#### Perineodynia (Table 5)

**Table 5:** Perineodynia before and after surgery

		Visual analog scale		Location		Worse while	
				
Patients	Follow-up (months)	Before	After	Before	After	Before	After
Ck	12	5	3	Left	Left	Sitting Walking Stairs	Sitting Stairs
Dz	18	0	0	-	-	-	-
Rx	12	7	2	Right	Right	Sitting Standing	Sitting
Re	19	8	0	Bilateral	0	Sitting Standing Defecation	0
Hy	39	3	3	Bilateral	Bilateral	Standing	Standing
Ro	11	8	2	Bilateral	0	Defecation Post-coital	Post-coital
Ns	12	0	0	-	-	-	-
Sn	12	4	2	Left	Left	Sitting	Sitting
Hs	10	4	0	Proctalgia fugax	0	-	-

The intensity of perineodynia (perineal pain), its location and its aggravating circumstances before and after surgery are presented in Table 5. Pain was cured in 2 patients, improved in 4 and was unchanged in one.

The summary of the effects of RLPM on perineodynia and on the urological, colo-proctologic and gynaecological axes is presented in Table 6.

**Table 6:** Summary of the effects of RLPM on the functional troubles of the perineum

Symptoms, Signs	Cured	Improved	Failure	Unknown
SUI	2	0	0	
Frequency	2	0	0	
Nocturia	1	0	0	
Urgency	3	0	2	
Dysuria	2	1	1	
Anal incontinence	2	1	0	
Dyschesia	6	1	1	
Cystocele	3	0	1	
Rectocele	4	0	1	
Dyspareunia	4	1	1	2 (no sex)
Perineodynia	2	4	1	

SUI = stress urinary incontinence.

### 3. Effect on the three clinical signs of pudendal neuropathy

The effects of RLPM on the 3 clinical signs of pudendal neuropathy are showed in Table 7. On the 12 positives with abnormal clinical signs, 5 were normalized by surgery and 7 did not change. On the 9 with negative (normal) clinical signs, 4 became abnormal after RLPM.

**Table 7:** Clinical signs of pudendal neuropathy before and after surgery

		Abnormal sensibility		Painful pudendal nerve		Positive skin rolling test	
				
Patients	Follow-up (months)	Before	After	Before	After	Before	After
Ck	12	1	1	1	1	0	1
Dz	18	0	1	0	0	0	0
Rx	12	-	-	-	-	-	-
Re	19	0	0	1	0	1	0
Hy	39	0	0	0	1	0	1
Ro	11	-	-	-	-	-	-
Ns	12	1	0	1	0	0	0
Sn	12	1	1	1	1	1	0
Hs	10	1	1	1	1	1	1

0 = normal (negative) test

1 = abnormal (positive) test

### 4. Effect on levator plate position and sagging

The objective evaluation of the position of the levator plate and anal margin at rest and during straining before and after RLPM is showed in Table 8.

**Table 8:** Objective evaluation of the levator plate and anal margin positions at rest and during straining before and after surgery

Patients	Perineocaliper (cm)						Retro-anal ultrasound (degrees)						Rectal examination (0–2)	
			
	Rest Before	Rest After	Strain Before	Strain After	Descent Before	Descent After	Rest Before	Rest After	Strain Before	Strain After	Sag Before	Sag After	Sag before	Sag after
Ck	-1	0	-3	-0,5	2	0,5	35	16	60	24	25	8	2	0
Dz	0	2	-1,5	1,5	1,5	0,5	-	-	56	20	-	-	1	0
Rx	-	-	-	-	-	-	-	-	47	25	-	-	2	1
Re	-1,5	-1	-2	-1,5	0,5	0,5	-	-	-	-	-	-	2	1
Hy	0,5	1	-1,5	0,5	2	0,5	31	22	58	29	27	7	2	1
Ro	-	-	-	-	-	-	29	25	53	35	24	10	2	1
Ns	-	-	-	-	-	-	24	17	56	28	32	11	2	0
Sn	1,5	0,5	-1	-0,5	2,5	1	28	31	43	43	15	12	2	1
Hs	0,5	0,5	-1	0	1,5	0,5	25	18	33	25	8	7	2	0
Mean	0	0.5	-1.66	-0.08	1,66	0,58	28,66	21,5	50.75	28.62	21.83	9,16	1.88	0.55

Evaluation of LPS by rectal examination after surgery showed a complete suppression of sagging in 4 patients and a reduction in the 5 others.

With the Perineocaliper^®^, the position of the anal margin at rest was 0.5 cm (extremes: -1–2) higher after surgery. During Valsalva's maneuver (strain), it was 1.58 cm (extremes: 0.5–3) higher. The reduction of perineal descent was therefore 1.08 cm (extremes: 0–1.5).

The mean reduction of angulation of the levator plate using retro-anal ultrasound at rest was 7.16 degrees (extremes: -3 – 19). During straining the mean reduction of angulation was 22.13 degrees (extremes: 0 – 36). The mean reduction of sagging was 12.67 degrees (extremes: 1 – 21).

### 5. Complication

In this case series, one patient (case "Ro") had a complete rupture of the RLPM during heavy lifting 12 months after surgery. Stress urinary incontinence, cystocele, rectocele, perineodynia after defecation and dyspareunia returned rapidly after the incident.

## Discussion

In perineology, only one specialist must treat all the symptoms of the three compartments of the perineum by using low risk and defect specific procedures.

By treating surgically only seven basic defects with their dedicated procedure it is possible to improve most of the functional troubles encountered in this area.

Abnormal levator plate sagging is one of these basic defects that must be treated in perineology [2]. This defect is quite frequent but rarely isolated.

The other ones are:

- relaxation of the sub-urethral vaginal hammock with hypermobility of the bladder neck (explaining mainly genuine stress urinary incontinence).

- rupture or weakness of the anterior part of the pelvic fascia – Halban's fascia (inducing cystocele).

- rupture or weakness of the posterior part of the pelvic fascia – Denonvilliers fascia, including its fixation to the utero-sacral ligaments

(favouring high rectocele, enterocele, uterine descent and cuff prolapse)

- weakness of the perineal body (increasing the risk of all prolapses but especially of low rectocele)

- rupture of the anal sphincter (with anal incontinence)

- pudendal neuropathy [20].

In the 104 patients treated by RLPM, only 9 (8,6%) had an isolated procedure and represent the studied population. In the 95 other cases, one or many of the six other defect specific procedures were done together to achieve a complete restoration of the perineum. These cases were excluded from the study because each of the associated procedures can have an important effect by itself on perineal function and anatomy.

Because this study focus on the defect called levator plate sagging, its first issue is to define when this sagging is abnormal. Three methods were used to evaluate this defect: the Perineocaliper^®^, rectal examination and retro-anal ultrasound.

In 1982, Henry et al studied the relationship of the anal verge to the ischial tuberosities in patients with descending perineum syndrome and compared them with normal subjects. They do not specify the name of the instrument used [26]. Some authors utilize the name "perineometer" for this instrument and perform perineometry with it [27]. Kegel had used this name many years earlier for an instrument that is introduced in the vagina to measure the increase of pressure induced by a perineal contraction [28]. The name "Perineocaliper" has been introduced because the measurement done in perineology corresponds to a depth or step measurement obtained with a Vernier caliper and the instrument has practically the same shape as a brake or skinfold caliper. With this instrument it is possible to define precisely the position of anal margin at rest, during Valsalva's maneuver or during pelvic floor contraction.

By using the Perineocaliper^® ^in a control group of 143 female patients, mean aged 55 years (extremes 26–81) without any symptoms, the normal position of the anal margin at rest was 0.03 cm above the ischial tuberosities (SD = 0.99) and during Valsalva 0.56 cm below these bones (SD = 0.98). The mean descent of the anal margin was 0.59 (SD = 0.54) (unpublished data). According to these data, a descent of more than 1.67 cm is unusual in a control group.

In a recent study, Beco [19,24] demonstrated that besides dyschesia and anal incontinence, a perineal descent of more than 1.5 cm measured with a Perineocaliper^® ^during Valsalva's maneuver in a gynaecological position significantly increases the frequency of urinary incontinence, dyspareunia, dysuria, cystocele and rectocele. The frequency of the 3 clinical signs of pudendal neuropathy [20] was also significantly increased (Table 9).

**Table 9:** Frequency of the 6 main perineological symptoms and of the 3 signs of pudendal neuropathy according to the perineal descent measured with a Perineocaliper^® ^[19,24].

**Perineal descent**	**Number of cases**	**Urinary incontinence**	**Faecal incontinence**	**Prolapse 2–3**	**Dysuria**	**Dyschesia**	**Dyspareunia**	**3 signs**
	**(n = 991)**	**(n = 566)**	**(n = 41)**	**(n = 256)**	**(n = 93)**	**(n = 261)**	**(n = 234)**	**(n = 152/820)**
**-1**	5	80	0	0	40	0	20	0
**-0,5**	7	42,85	0	42,85	0	14,28	14,28	0
**0**	227	51,54	1,32	21,58	10,13	24,22	14,53	13,87
**0,5**	257	50,19 (NS)	3,50 (NS)	20,62 (NS)	6,22 (NS)	15,95 (NS)	17,89 (NS)	17,28 (NS)
**1**	308	60,06 (p<0.05)	4,54 (p<0.05)	25 (NS)	8,76 (NS)	25,97 (NS)	27,59 (p<0.001)	18,41 (NS)
**1,5**	76	60,52 (NS)	3,94 (NS)	34,21 (p<0.05)	9,21 (NS)	35,52 (NS)	32,89 (p<0.001)	23,33 (NS)
**2**	82	75,60 (p<0.001)	12,19 (p<0.001)	43,90 (p<0.001)	13,41 (NS)	48,78 (p<0.001)	36,58 (p<0.001)	27,94 (p<0.01)
**2,5**	15	66,66	6,66	33,33	20	53,33	46,66	55,55
**3**	11	81,81	9,09	54,54	27,27	72,72	54,54	44,44
**3,5**	2	50	0	50	50	50	0	0

The p values were obtained using chi-squared tests; comparison with descent = 0. A perineal descent of 2 cm (compared to a 0 cm descent) leads to a significant increase in the frequency of urinary incontinence, faecal incontinence (solid stools), genital prolapse (grade 2 and more) and dyschesia. The same threshold exists for the 3 clinical signs of pudendal neuropathy (820 cases). For dyspareunia the threshold seems to be at 1 cm and in case of dysuria, the difference is significant between 0.5 cm and 2 cm of descent (p < 0.05).

Therefore, it seems that a perineal descent of more than 1.5 cm during a Valsalva's maneuver is abnormal.

Henry [26], using an old version of the same instrument, found different values in his control group of 55 women (mean age 48 years): at rest + 2.5 cm (SD = 0.6), during "bears down" like in defecation + 0.9 cm (SD = 1). There are three main differences between the two studies: the mean age of the control group (48 versus 55 years), the position of the patient during the measurement (left lateral versus gynecological position) and the type of effort ("bears down" like in defecation versus Valsalva's maneuver). They could explain the different results obtained.

Measuring the descent of the anal margin is an indirect method of evaluating levator plate sagging. In fact, theoretically the descent of the anal margin is greater in cases of a longer levator plate for the same angle. Therefore it seems logical to evaluate the angle itself.

This evaluation is possible by rectal examination as proposed by Shafik [15]. The three levels ordinal scale used in this study was very useful in the indication of RLPM (grade 2 = complete sagging) and at the end of the myorrhaphy (grade 0 = no sagging). Other methods like colpocystodefecography, magnetic resonance imaging or ultrasound must be used to obtain more precise data.

For Costalat et al [29] using colpocystodefecography in a sitting position, the normal angulation of the posterior rectal wall at rest is less than 20 degrees with the horizontal. During defecation, the angle of sagging must be less than 20 degrees.

For Hsu et al [30] using dynamic nuclear resonance magnetic imaging in a supine position, the mean angulation of the levator plate with the vertical plane at rest was 36.2 degrees (SD = 12.3) and during Valsalva's maneuver 44.3 degrees (SD = 15.2). With respect to the control group, there was a significant increase of 9.1 degrees in the levator plate angle during Valsalva in case of prolapse.

Retro-anal ultrasound (done in a gynecological position) is a new easily available method that enables the study of the levator plate angle. In a control group of 40 female patients (unpublished data), with a mean age of 51 years (extremes: 23–81) the mean value of the angles at rest was 19.7 degrees (SD = 8.8 degrees) and during Valsalva 30.5 degrees (SD = 10.7 degrees). The mean sagging angle (difference between Valsalva and rest angles) was 10.8 degrees (SD = 8.2 degrees). This method has been used to evaluate the changes in angles induced by surgery, but not as an inclusion criteria for surgery because it is still being validated.

Anti-sagging tests must be used to prove that perineal descent is the cause of one symptom. By supporting the posterior perineum, these tests suppress levator plate sagging and simulate the effect of RLPM. It must be used to confirm the indication to perform a RLPM, but it may also be used as a part of the non-surgical treatment of a descending perineum syndrome. Especially in case of dyschesia or dysuria, the improvement of micturition or defecation by moving back as much as possible on the toilet seat can be so important that it can stop the vicious circle: straining => perineal descent (stretching of the pudendal nerve) => straining. Ideally, the back part of the toilet seat must be large enough and only slightly tilted to support perfectly the posterior perineum. The same approach was proposed by Lesaffer [17,18] and Nichols [14,16] who suggest the use of special toilet seats or supports to improve defecation.

Overload of the "suspensor structures" (pubo-rectalis, utero-sacral ligaments, vaginal scars, transverse muscles) induced by levator plate sagging is a newly discovered cause of perineodynia and dyspareunia which must be differentiated from pudendal neuropathy [31] or muscular pain [32]. Contrary to the two other causes, pain induced by levator plate sagging is usually increased while standing and reduced while sitting. It is suppressed by the anti-sagging test. This test is also very helpful to confirm that perineal descent is the cause of dyspareunia.

The same differential diagnosis must be done in cases of urinary frequency, urgency and urge incontinence where pudendal neuropathy [20,33] and muscular trigger points [34] may also be involved. Again, in cases of levator plate sagging, the symptoms are worse in a standing position and disappear when the posterior perineum is lifted with the hand.

Some surgical procedures able to reduce levator plate sagging have been described in the literature. A comparison between these procedures and RLPM is presented in Table 10.

**Table 10:** Surgical procedures used to treat levator plate sagging

	Post-anal repair (Parks [6])	Retro-rectal levatorplasty (Nichols [14,16])	Levatorplasty (Shafik [15])	Retro-anal levator plate myorrhaphy
Retro-anal incision	U-shaped	Midline	U-shaped	Midline
Dissection plane	Intersphincteric	Retro-anal	Retro-anal	Retro-anal
Opening of the pelvis	Incision of Waldeyer's fascia	Opening of the retro-rectal or pre-sacral space	No	No
Myorrhaphy	Levator plate, pubo-rectalis and external sphincter	Levator plate and pubo-rectalis	Levator plate	Levator plate
Rectal neck attached to the levator plate	No	No	Yes	No
Posterior wall of the rectum sewn to the presacral fascia	No	Yes	No	No

The intersphincteric plane has been avoided because this approach can damage the thin anal sphincter and the operating field is reduced. After having used retro-anal U-shaped skin incision (Shafik's levatorplasty [15]) in our first cases (not included in this case series), we decide to make midline skin incisions which are less painful. Only myorrhaphy of the levator plate has been done because the pubo-rectalis is included in the upper part of the anal sphincter and is not available for suture by the retro-anal approach. Furthermore, stitches in this muscle would damage it and therefore reduce its contractile force. To reduce the risk of injury to the rectum, the rectal neck was not attached to the edge of the levator plate (this step is probably necessary in cases of rectal prolapse [15]) and the pelvis was not opened.

In the literature, the main indication of post-anal repair was idiopathic anal incontinence. The long term results vary from 90% cured in the old studies to 35% cured in the more recent ones [7-10]. Nichols [14] used retro-rectal levatorplasty to treat isolated dyschesia with anal descent. Shafik [15] proposed levatorplasty to treat dyschesia associated with complete rectal prolapse.

In this short case series, besides anal incontinence and dyschesia, RLPM has been effective on most of the functional troubles of the perineum. Of course the number of cases is too small to draw definitive conclusions. This new solution may though be very useful to treat difficult problems such as urinary urgency, dysuria, dyspareunia or perineodynia. For stress urinary incontinence, RLPM is not a first line treatment because less invasive and more specific procedures exist but it could be helpful in some difficult cases.

Athanasiadis [9] found no significant difference in pelvic descent and ano-rectal angle after post-anal repair. After the same surgical procedure, Orrom [11] and Van Tets [12] did not find any difference either in the ano-rectal angle. In this study, levator plate sagging was reduced independently from the evaluation method used. This difference may be due to the smaller number of cases, to a shorter follow-up or to the different surgical approach.

Jameson [8] and Athanasiadis [9] didn't find any significant change in the motor latencies of the pudendal nerve after post-anal repair. In this study, the effect on pudendal neuropathy is unclear. By reducing the stretching on the pudendal nerve, RLPM should normally improve pudendal nerve function. This result has been observed clinically in 50% of the cases. The appearance of some clinical signs of pudendal neuropathy after surgery is quite surprising. Maybe it is linked to the formation of adhesions in the ischio-rectal fossae or the appearance of trigger points in the pelvic floor muscles. Further studies are necessary to better understand this side effect.

RLPM is one defect specific treatment of levator plate sagging, but of course other surgical procedures could reduce perineal descent as well. The patient with rupture of the RLPM has been re-operated successfully. The procedure used was part of a full defect restoration project [2] and included the treatment for rectocele, cystocele and urinary incontinence using the vaginal route. Without any statistical proof, this multi-layer operation seems to be more logical to reduce the load on the RLPM. In fact each layer (anterior vaginal wall, sub-urethral support, posterior vaginal wall, perineal body) absorbs a part of the pressure, therefore reducing the tension on the levator plate myorrhaphy. Conversely, RLPM should reduce the load on genital prolapse repairs and the risk of recurrence.

In our full experience with RLPM, the main risk is ischio-rectal fossae infection (not encountered in this case series). This risk seems to be reduced by the use of a multi-tubular drain for 5 days, antibiotic coverage and intestinal preparation before surgery.

## Conclusion

Dyschesia and anal incontinence are only a small part of the problems induced by pathological sagging of the levator plate. In fact, perineal descent of more than 1.5 cm significantly increases the frequency of all the functional troubles related to the perineum.

RLPM can treat levator plate sagging, perineal descent and all the symptoms associated with this defect (stress urinary incontinence, frequency, urgency, dysuria, anal incontinence, dyschesia, dyspareunia, perineodynia and prolapse). Anti-sagging tests must be used to confirm the role of this sagging in the origin of these symptoms. A clear positive test is mandatory before surgery is indicated. Sometimes though, simply moving back on the toilet seat during defecation and micturition is enough to treat the patient's problem, avoiding the need for physiotherapy or surgery.

Many symptoms induced by levator plate sagging may be seen in cases of pudendal neuropathy or pelvic floor muscle trigger points. The perineology specialist must be aware of all these etiologies to obtain good results for his patients.

## Abbreviations

RLPM: retro-anal levator plate myorrhaphy; LPS: levator plate sagging; DPS: descending perineum syndrome; SD: standard deviation; SUI: stress urinary incontinence.

## Competing interests

The author has designed the instrument used to measure perineal descent and is the owner of the registered mark Perineocaliper^®^. The society which sell this instrument did not participate financially to this study.

## Authors' contributions

JB carried out all of the work for this study.

## Pre-publication history

The pre-publication history for this paper can be accessed here:


